# Asymmetric electrostatic dodecapole: compact bandpass filter with low aberrations for momentum microscopy

**DOI:** 10.1107/S1600577524003540

**Published:** 2024-06-20

**Authors:** O. Tkach, S. Chernov, S. Babenkov, Y. Lytvynenko, O. Fedchenko, K. Medjanik, D. Vasilyev, A. Gloskowskii, C. Schlueter, H.-J. Elmers, G. Schönhense

**Affiliations:** a Institut für Physik Johannes Gutenberg-Universität Mainz 55099Mainz Germany; b Sumy State University Rymskogo-Korsakova 2 Sumy40007 Ukraine; c Deutsches Elektronen-Synchrotron DESY 22607Hamburg Germany; d Institute of Magnetism of the NAS and MES of Ukraine Kyiv03142 Ukraine; ESRF – The European Synchrotron, France

**Keywords:** momentum microscopy, bandpass prefilters, HARPES, hard X-ray angle-resolved photoelectron spectroscopy, dodecapoles, photoelectron diffraction

## Abstract

Imaging energy filters in photoelectron microscopes and momentum microscopes use spherical fields with deflection angles of 90°, 180° and even 2 × 180°. These instruments are optimized for high energy resolution, and exhibit image aberrations when operated in high transmission mode at medium energy resolution. Here, a new approach is presented for bandpass-filtered imaging in real or reciprocal space using an electrostatic dodecapole with an asymmetric electrode array. In addition to energy-dispersive beam deflection, this multipole allows aberration correction up to the third order. Here, its use is described as a bandpass prefilter in a time-of-flight momentum microscope at the hard X-ray beamline P22 of PETRA III. The entire instrument is housed in a straight vacuum tube because the deflection angle is only 4° and the beam displacement in the filter is only ∼8 mm. The multipole is framed by transfer lenses in the entrance and exit branches. Two sets of 16 different-sized entrance and exit apertures on piezomotor-driven mounts allow selection of the desired bandpass. For pass energies between 100 and 1400 eV and slit widths between 0.5 and 4 mm, the transmitted kinetic energy intervals are between 10 eV and a few hundred electronvolts (full width at half-maximum). The filter eliminates all higher or lower energy signals outside the selected bandpass, significantly improving the signal-to-background ratio in the time-of-flight analyzer.

## Abstract 2

A compact bandpass prefilter eliminates electrons with energies above or below the desired range and can correct image aberrations up to the third order before the beam enters a time-of-flight analyzer. Here, the imaging performance of the filter is demonstrated for two key applications of high-energy momentum microscopes: full-field core-level photoelectron diffraction and mapping of bulk valence bands.

## 1. Introduction

Imaging bandpass filters play an important role in photoelectron spectroscopy. Such filters can transport either real-space images, as studied in detail by Tonner (1990 ▸), or momentum-space images, as pioneered by Krömker *et al.* (2008 ▸). The latter family of instruments established a new type of angle-resolved photoelectron spectroscopy (ARPES) called momentum microscopy. This early work initiated the development of several types of momentum microscopes (MMs), whose front lenses convert the photoelectron angular distribution into a full-field image of the transversal (*k*_*x*_, *k*_*y*_) momentum pattern. Dispersive-type MMs use either a tandem arrangement of two hemispherical analyzers (Krömker *et al.*, 2008 ▸; Escher *et al.*, 2005 ▸; Winkelmann *et al.*, 2012 ▸; Tusche *et al.*, 2015 ▸, 2019 ▸) or a single hemispherical analyzer (Schönhense *et al.*, 2020*a* ▸, 2021*a* ▸; Tesch *et al.*, 2018 ▸; Matsui *et al.*, 2020 ▸). These instruments are similar to the spectroscopic low-energy electron microscope (SPELEEM) developed by Bauer and coworkers (Schmidt *et al.*, 1998 ▸; Locatelli *et al.*, 2006 ▸). An alternative design using the dispersive power of a magnetic prism has been developed by Tromp *et al.* (2009 ▸).

In all of these instruments, energy-filtered 2D images (real or reciprocal space) are transported through the analyzer using concepts from charged-particle optics and electron microscopy. As in conventional spectrometers, the energy resolution is defined by the pass energy and the size of the entrance and exit slits. A resolution of 4.2 meV has been achieved using a single-hemisphere setup (Schönhense *et al.*, 2021*a* ▸). Another type of MM is based on time-of-flight (ToF) recording (Medjanik *et al.*, 2017 ▸). ToF-MMs simultaneously acquire an energy band of several electronvolts width using a time-resolving image detector, allowing 3D (*E*_kin_, *k*_*x*_, *k*_*y*_) recording.

The hemisphere-based instruments have one or two 180° deflections (Tonner, 1990 ▸; Krömker *et al.*, 2008 ▸; Escher *et al.*, 2005 ▸; Winkelmann *et al.*, 2012 ▸; Tusche *et al.*, 2015 ▸, 2019 ▸; Schönhense *et al.*, 2020*a* ▸, 2021*b* ▸; Tesch *et al.*, 2018 ▸; Matsui *et al.*, 2020 ▸) or one 90° deflection (Schmidt *et al.*, 1998 ▸; Locatelli *et al.*, 2006 ▸) with corresponding transfer lens systems. Thus, these instruments are associated with image aberrations when they are operated in high transmission mode with medium energy resolution. Here, we present a new design of a compact electrostatic bandpass filter that fits into the linear column of a ToF-MM. We adopt the concept of an electrostatic dodecapole, as theoretically studied in detail by Boerboom and coworkers (Boerboom *et al.*, 1985 ▸; Matsuo *et al.*, 1982 ▸). This type of multipole allows the superposition of dipole, quadrupole and hexapole fields, and thus can correct image aberrations up to the third order.

A prototype dodecapole is used as a prefilter in a ToF-MM at the hard X-ray beamline P22 at PETRA III (Schlueter *et al.*, 2019 ▸), where it significantly reduces the background signal and eliminates artefacts in the ToF spectra. At high kinetic energies, the ‘temporal aliasing’ effect (Medjanik *et al.*, 2019 ▸) can lead to artefacts in spectra and momentum patterns. These artefacts are caused by electrons whose ToF differs from the flight time of the probe electrons of interest by a multiple of the pulse period of the photon source. The time-resolving detector cannot distinguish such (faster or slower) background electrons from the true signal. The energy-dispersive bandpass filter limits the recorded energy interval to the region of interest, thus completely eliminating the aliasing artefacts.

The dodecapole arrangement consists of an entrance and an exit branch, both of which contain sets of 16 size-selectable and position-adjustable apertures that serve as entrance and exit apertures. The transmitted energy band depends on the pass energy, slit width and deflection angle. When used for pre-filtering, the dodecapole unit must not be operated with a high energy resolution, as the desired final resolution is reached by the ToF analysis. For the prototype at beamline P22, a small deflection angle of only 4° was chosen. This allows the use of a linear vacuum housing with mumetal tube for magnetic shielding.

## 2. The asymmetric dodecapole: a versatile device for energy filtering and aberration correction

### 2.1. Motivation for the development of the dodecapole filter

The motivation for the development of the dodecapole with asymmetrical electrode arrangement originated from an inherent problem of the ToF method. ToF analyzers act as high-pass filters: all electrons with energies above a certain cutoff, defined by the potential of the drift tube, can pass the ToF section and reach the detector. The time-resolving detector is triggered by the bunch clock of the pulsed source and the trigger pulses come in with the period *T* of the photon pulses. Hence, the detector cannot distinguish electrons with ToF τ, τ + *nT* or τ − *nT* (*n* is a natural number). The period *T* is thus the crucial quantity. The majority of storage rings use filling patterns with *T* = 2 ns (500 MHz); a few, *e.g.* MAX IV, have *T* at 10 ns (100 MHz). Special filling patterns are provided by PETRA III in 40-bunch mode with *T* = 183 ns (∼5 MHz) or BESSY-II in single-bunch mode with 1 µs (1 MHz). The FELs FLASH and European XFEL, both hosting ToF-MMs, provide micro-bunch trains with 1 MHz. Laboratory sources range between 16 ns (60 MHz) for intra-cavity enhanced high-harmonic sources (Kunin *et al.*, 2023 ▸) and the ∼10 µs range (∼100 kHz) (*e.g.* Tkach *et al.*, 2024 ▸). The ToF τ of the electrons in the ToF analyzer can exceed the period *T*. Fast electrons released by the pulse at *t*_0_ can overtake slow electrons from earlier pulses at *t*_0_ − *nT*. This temporal aliasing effect is quantified for realistic conditions in the hard X-ray range in Fig. 4(*f*) of Medjanik *et al.* (2019 ▸).

**Figure 1:**
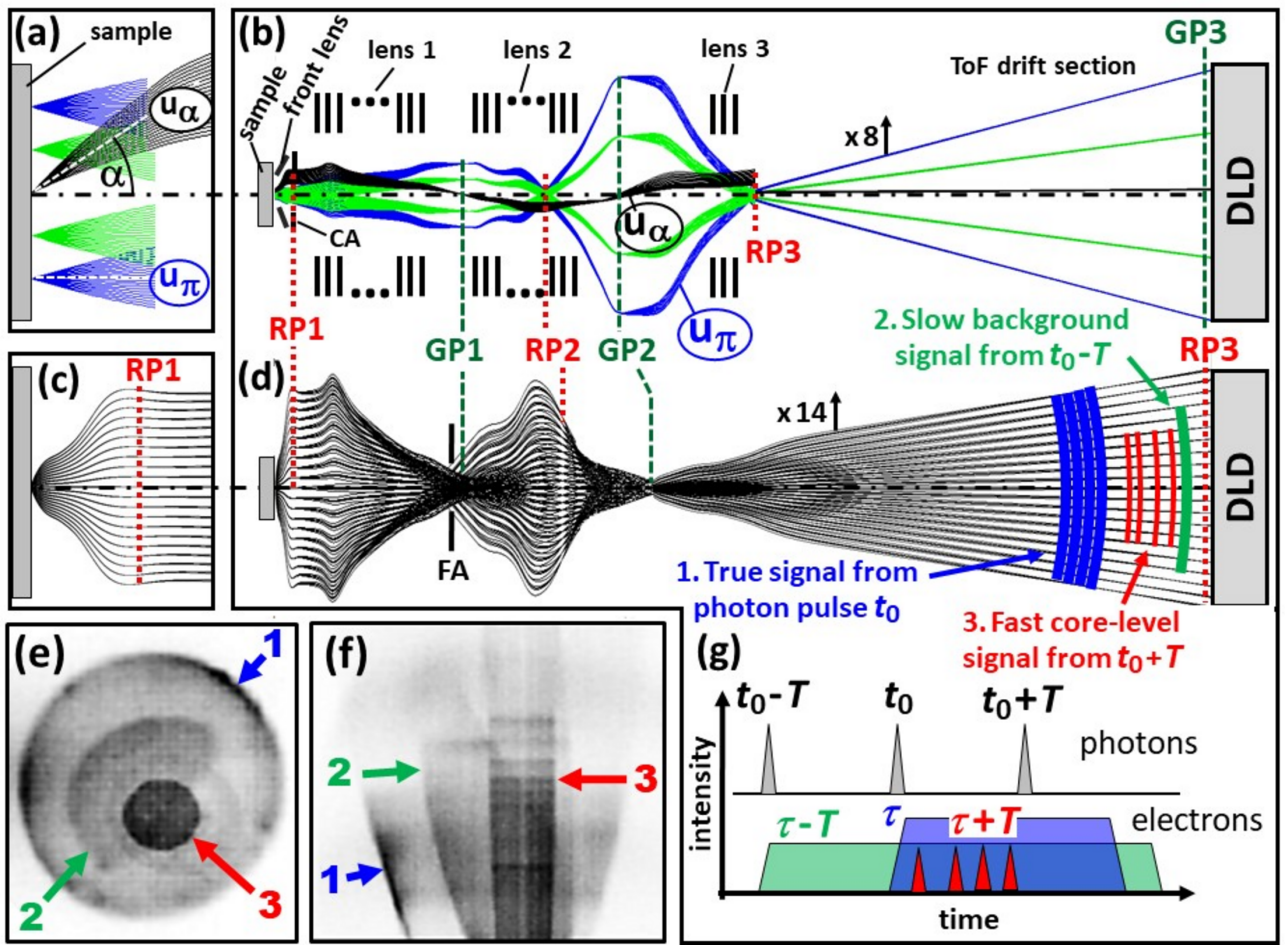
Working principle of a ToF photoelectron microscope, demonstrated by ray-tracing calculations for a typical microscope geometry. (*a*, *b*) ToF-PEEM mode, illustrated by ray bundles around the fundamental rays u_α_, starting on the axis at an angle α, and u_π_, starting parallel to the optical axis, as seen in the close-up view (*a*); the angles are greatly exaggerated. Lenses 1, 2 and 3 are multi-element groups, as schematically sketched; stigmators and deflectors are omitted. RP1, RP2 and RP3 (red dotted lines) are the reciprocal image planes; GP1, GP2 and GP3 (green dashed lines) are the Gaussian (real space) image planes; and DLD is the delay-line detector. (*c*, *d*) Same as (*a*, *b*), but for the momentum-microscopy mode. The first *k* image appears in the plane RP1 (backfocal plane of the front lens), marked in (*c*). In (*b*) and (*d*), the ToF drift sections are radially compressed by factors of 8 and 14, respectively. (*e*) *k*_*x*_–*k*_*y*_ and (*f*) τ vs *k***_∥_** sections of a measured momentum pattern with three overlapping signals due to the temporal-aliasing effect. The largest pattern (1) corresponds to the electrons of interest with ToF τ. Patterns 2 and 3 result from slower and faster electrons with ToFs of τ + *T* and τ − *T*, respectively, where *T* is the period of the photon pulses. A series of fast core-level photoelectrons is visible as horizontal stripes in signal 3. (*g*) Intensity vs time schemes for photons and electrons (see main text for details). (Ray tracing using *SIMION* 8.0; Dahl *et al.*, 2007 ▸.)

To understand how the dodecapole eliminates this effect, we first need to examine the functioning principle of a ToF-MM. Figs. 1 ▸(*a*) and 1 ▸(*b*), and 1 ▸(*c*) and 1 ▸(*d*) display the ToF photoemission electron microscopy (ToF-PEEM) mode and the ToF momentum microscopy mode, respectively. Figs. 1 ▸(*a*) and 1 ▸(*b*) show the two fundamental rays: the axial ray u_α_, which starts on the axis at an angle α, and the field ray u_π_, which runs parallel at a distance from the optical axis. To enhance visibility, we display ray bundles around u_α_ in black and around u_π_ in blue. Additionally, a green field ray is shown at half the distance from the axis. The first *k* image is formed in the backfocal plane (also known as the reciprocal plane RP1) of the front lens, as detailed in Fig. 1 ▸(*c*). The front lens and lens 1 create a real-space image of the sample in the first Gaussian plane GP1. The u_α_ ray intersects with the axis, while the field rays (blue and green) run parallel to the optical axis, determining the lateral magnification. The zoom lens 2 can adjust the magnification between Gaussian planes GP1 and GP2, and lens 3 produces the final PEEM image in GP3 [at the delay-line detector (DLD)]. To enhance resolution and contrast, a contrast aperture (CA) can be inserted in plane RP1 [top left of Fig. 1 ▸(*b*)]. The low-energy drift section, necessary for energy dispersion, ensures a large final magnification. The simulation provided is for a realistic lens system with a 200 µm field of view [blue rays in Fig. 1 ▸(*a*)] and magnifications of *M*_1_ = 11, *M*_2_ = 42 and *M*_3_ = 330 in GP1, GP2 and GP3, respectively.

In the *k*-imaging mode, Figs. 1 ▸(*c*) and 1 ▸(*d*), the region on the sample is typically an order of magnitude smaller, either due to a small photon spot or due to the confinement of the analyzed region by a field aperture (FA) in GP1, see Fig. 1 ▸(*d*). However, the observed *k* range is much larger than in PEEM mode. The zoom lens 2 and transfer lens 3 are adjusted so that the final reciprocal plane RP3 is shifted towards the detector. At a given time *t*, there is a certain distribution of electrons on their way to the detector. The colored contours of the isochrones mark three different types of electrons: (1) the signal of interest (released by the photon pulse at *t*_0_), (2) the slow electron background (from the previous pulse *t*_0_ − *T*) and (3) fast core-level photoelectrons from the next pulse (*t*_0_ + *T*) or from core levels with smaller binding energy. Faster electrons often originate from higher-order admixtures in the photon beam. These fast core-level electrons are underfocused, while the slow background electrons are overfocused, both of which result in a smaller diameter of the *k* pattern.

Figs. 1 ▸(*e*) and 1 ▸(*f*) show an experimental example measured at beamline P22 at PETRA III (DESY, Hamburg) at *h*ν = 4 keV. It shows the expected behavior. The different species 1, 2 and 3 appear with different diameters in the *k*_*x*_–*k*_*y*_ pattern [Fig. 1 ▸(*e*)]. The τ vs *k*_||_ section [Fig. 1 ▸(*f*)] shows the low-energy electrons with their typical parabolic rim and the fast core-level electrons as a band with parallel rims. A series of core levels appear as horizontal stripes. Fig. 1 ▸(*g*) shows a scheme of the temporal structure for this case. The blue, green and red signals fall in the same time interval, although they originate from different photon pulses and have different energies. This case with two parabolic rims is rather an exception.

In summary, faster and slower electrons can be detected in the same time interval as the true signal, if their ToF differs from that of the true electrons by a multiple of *T*. This can lead to significant artefacts in the momentum distributions because the time-resolving detector is unable to distinguish these false ‘modulo *T*’ electrons from the true signal. The only way to eliminate these background signals is to implement a dispersive energy filter. It has been demonstrated that a hemispherical analyzer can accomplish this task (Schönhense *et al.*, 2021*a* ▸). However, the electrons traveling in the spherical field experience significant time lags that require correction. The goal of this study was to create a compact dispersive bandpass filter with minimal aberrations that does not affect the time structure of the electron signals.

### 2.2. The asymmetric dodecapole as a versatile electron-optical element

We have chosen the dodecapole as a dispersive prefilter because it can generate a number of multipole fields for aberration correction up to the third order. The particle-optical properties of 12 electrodes in a dodecagon configuration have been studied in detail by Boerboom and coworkers (Boerboom *et al.*, 1985 ▸; Matsuo *et al.*, 1982 ▸). The most important features are: deflection in two perpendicular directions by dipolar fields, quadrupole focusing and stigmatization, correction of second- and third-order aberrations, and two-directional focusing. A unique advantage is that several of these applications can be affected simultaneously by superposition of multipole field arrays. First-, second- and third-order focusing can be independently controlled by appropriate settings of the voltages applied to the electrodes. The influence of the fringing field is of the fourth order and is thus disregarded in the third-order approximation.

**Figure 2:**
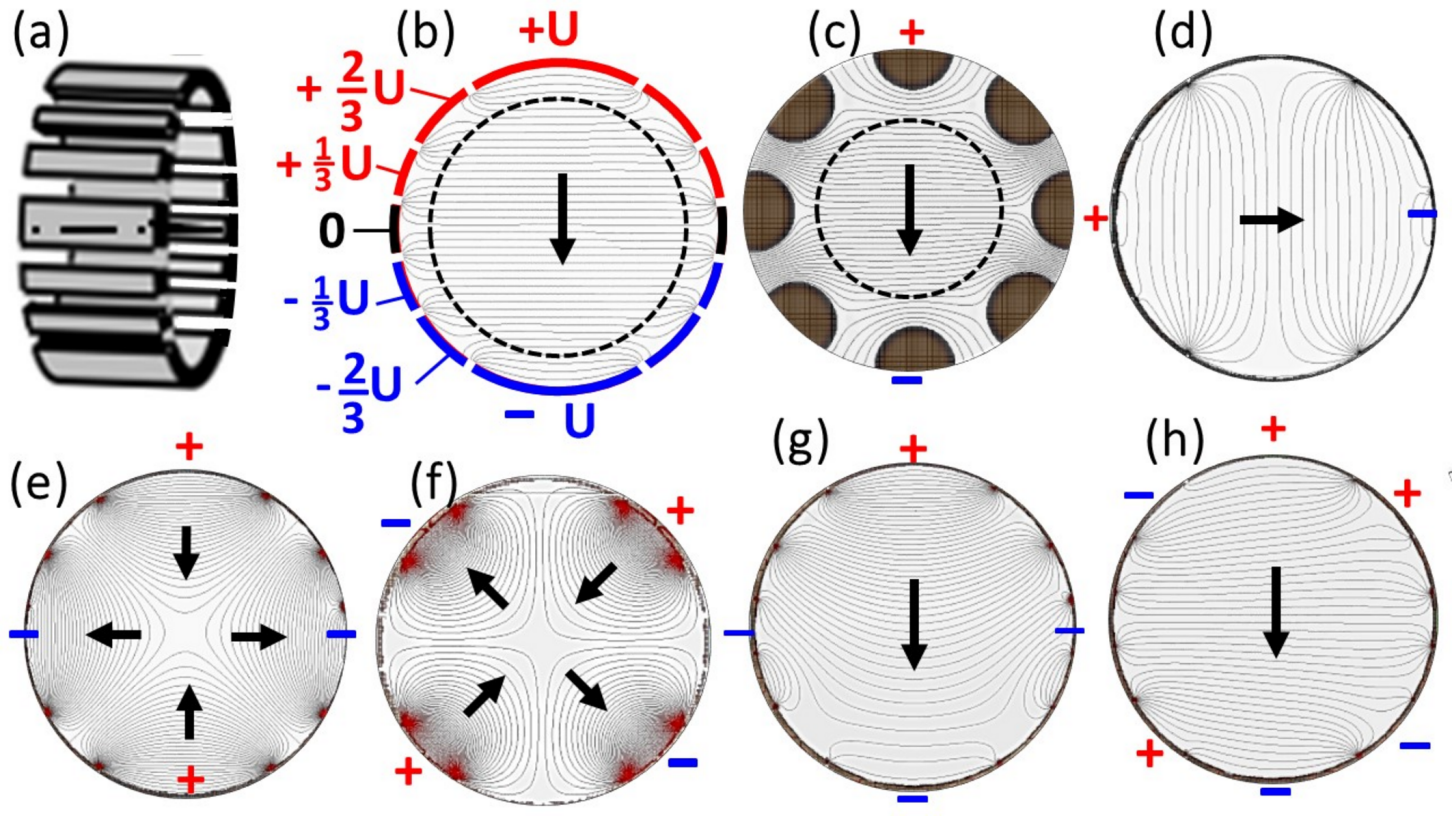
(*a*) A schematic view of the dodecapole bandpass filter. (*b*) Electrode geometry, voltages and calculated potential distribution in the center cross-sectional plane of the dodecapole; the dispersive plane is vertical. The arrow denotes the deflecting (dipole) field, which causes the energy dispersion. (*c*) The dipole field created by a conventional octupole deflector/stigmator with the same outer dimension as (*b*), shown for comparison. (*d*)–(*h*) Correcting multipole fields: (*d*) a horizontal dipole field correcting for beam position, and quadrupole fields oriented at (*e*) 0°/90° and (*f*) ±45° correcting for astigmatism. (*g*) and (*h*) Examples for the superposition of the deflector field (*b*) and the quadrupole fields [(*e*) and (*f*)], respectively.

Fig. 2 ▸(*a*) shows a schematic view of the electrode arrangement. Depending on the size of the recorded *k* field of view and the pass energy, the beam diameter in the momentum image located in the center of the dodecapole can be very large. Therefore, its geometry has been chosen such that the deflecting field is homogeneous in a range as large as possible. Otherwise the *k* image would be distorted upon deflection. Fig. 2 ▸(*b*) shows the asymmetrical arrangement and voltages of the electrodes with the center as the zero reference. The electrode geometry has been designed such that for a pure deflection field the voltages vary linearly: *U*, 2/3 *U*, 1/3 *U*, 0, −1/3 *U*, −2/3 *U*, −*U*. The calculated equipotential contours reveal that the field along the dispersive direction (vertical) is homogeneous in a large area of ∼80% of the total cross section (marked by the dashed circle). For comparison, Fig. 2 ▸(*c*) shows the electric field in a conventional octupole unit with the same outer dimensions as in Fig. 2 ▸(*b*), assuming that the centers of the rods lie on the outer contour of the unit. Here, the homogeneous region is much smaller, ∼50% of the total cross section.

Fig. 2 ▸ shows the potential distributions of the two perpendicular dipolar fields [Figs. 2 ▸(*b*) and 2 ▸(*d*)] and the two quadrupole fields [Figs. 2 ▸(*e*) and 2 ▸(*f*)], rotated by 45° with respect to each other. These have the function of (weak) cylinder lenses with selectable azimuthal orientation, compensating for astigmatism. Figs. 2 ▸(*g*) and 2 ▸(*h*) show equipotential lines for the superposition of the primary dipole field [Fig. 2 ▸(*b*)] with the differently oriented quadrupole fields [Figs. 2 ▸(*e*) and 2 ▸(*f*), respectively].

### 2.3. Properties of the dodecapole bandpass filter

In the limit of small deflection angles, the homogeneous field [marked by the arrow in Fig. 2 ▸(*b*)] deflects the electrons along parabolic trajectories. We have performed ray-tracing calculations in order to elucidate the resolving power for different geometries and deflection angles. Systematic simulations showed that an impact angle of 2° with respect to the symmetry axis of the dodecapole is a good compromise between sufficient resolution (when used as a bandpass prefilter) and small aberrations. For such a small angle, the fringing field effects at the entrance and exit of the dodecapole are negligible.

**Figure 3:**
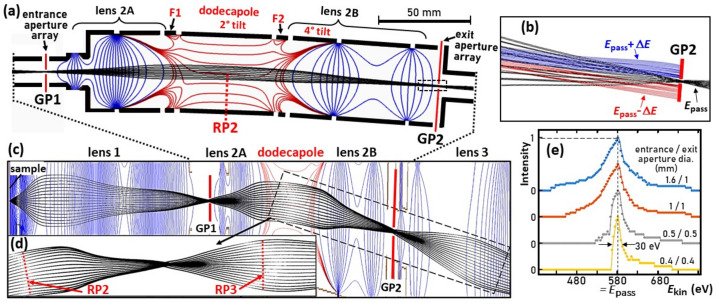
Ray-tracing simulations of a full 3D model of the dodecapole bandpass filter, integrated into the lens system of the ToF-MM at beamline P22 of PETRA III. (*a*) A scaled view (scale bar is 50 mm) of the bandpass filter, consisting of entrance aperture (in plane GP1), lens 2A, fringe field correctors F1 and F2 framing the dodecapole, lens 2B and exit aperture (plane GP2). Equipotential contours for the lens fields are in blue and for the dodecapole are in red. (*b*) A close-up view of the region near the exit aperture for five different energies: *E*_kin_ = *E*_pass_ = 580 eV (black rays) and faster (blue rays) and slower electrons (red rays) with Δ*E* varied in steps of 40 eV. (*c*) A view including lens 1 and lens 3 with the radial coordinate stretched. The dashed rectangle marks the region shown in (*d*) after dewarping (conformal scale). (*e*) A series of calculated transmission profiles; see the main text for details. (Ray tracing using *SIMION* 8.0; Dahl *et al.*, 2007 ▸.)

Fig. 3 ▸ displays a selection of results for the α = 4° geometry of the instrument installed at beamline P22. The assignment of lenses and planes is consistent with Fig. 1 ▸. The dodecapole is placed between the Gaussian planes GP1 and GP2. The dodecapole unit with entrance and exit slits and transfer lenses, shown in Fig. 3 ▸(*a*), replaces lens group 2. The FA serves as the entrance aperture, and an identical array of 16 apertures in GP2 serves as the exit aperture. This entrance aperture is equivalent to the FA in Fig. 1 ▸ and has two additional functions. First, it selects the diameter of the observed region of interest on the sample, independent of the size of the illuminating photon beam. Second, in an MM, the FA has the function of a contrast aperture for the *k* image. The transfer lens 2A produces a *k* image with parallel rays in the center of the dodecapole (reciprocal plane RP2). Lens group 2B focuses an image from plane GP1 into the plane of the exit aperture GP2. The dodecapole is tilted by 2° and the exit branch is tilted by 4°. The red equipotential contours (equally spaced between the upper and lower electrodes) show that the homogeneous field in this section has significant distortions at the entrance and exit of the dodecapole. Two additional electrodes F1 and F2 ensure that the field penetration of the lenses 2A and 2B is symmetrical at the entrance and exit. The focal spot in plane GP2 shows no significant distortions.

Fig. 3 ▸(*b*) shows seven groups of rays with different kinetic energies (inside of the dodecapole). Electrons with a kinetic energy of *E*_kin_ = 580 eV (= pass energy *E*_pass_) are focused to pass through the exit aperture (black rays). Three faster groups at 620, 660 and 700 eV (blue) and three slower groups at *E*_kin_ = 540, 500 and 460 eV (red) are deflected too weakly and too strongly, respectively. At Δ*E* = 40 eV (7% of *E*_pass_), all groups are completely separated. The dispersion in the exit plane is 5 µm eV^−1^. Fig. 3 ▸(*c*) shows a simulation for a *k*-field diameter of 10 Å^−1^ with a stretched radial coordinate, giving the appearance of an increased angle. Fig. 3 ▸(*d*) shows the detail marked as a dashed rectangle in Fig. 3 ▸(*c*) after dewarping, *i.e.* with a true 4° deflection angle. The pattern shows that the field curvature is increased in the *k* image in plane RP3 behind the exit aperture. However, there is no visible deformation of the *k* image due to the dodecapole in the dispersive plane. The dodecapole field acts as a weak cylinder lens, compressing the pattern in the dispersive plane by about 7% compared with the non-dispersive plane, which can be corrected by image processing.

Fig. 3 ▸(*e*) shows a series of simulations of transmission profiles for various aperture sizes [FA/SA = 1.6/1.0, 1.0/1.0, 0.5/0.5 and 0.4/0.4; all in millimetres]. The profiles were calculated by running 5000 rays for each curve with energy steps of 5 eV and emission-angle steps of 0.5°. The intensity histograms display the fraction of transmitted number of rays as a function of kinetic energy *E*_kin_. The maxima are normalized to 1. There may be beam clipping at the entrance aperture in GP1, depending on the aperture size and the photon footprint on the sample. The profiles are nearly symmetrical with a full width at half-maximum (FWHM) of ∼70 eV for large apertures. However, at 0.5 mm, the low-energy side is steeper, and at 0.4 mm, it shows a sharp cutoff and a FWHM of ∼30 eV. For small apertures, *i.e.* high resolution, the transmission profile shows an asymmetric shape with a steep slope at the low-energy end and a longer tail at high energies.

As in any electron spectrometer, the energy bandpass depends on the pass energy *E*_pass_ and the sizes of entrance and exit apertures. When using the instrument as a prefilter in a ToF-MM, we usually set the bandpass to 5–10%. In the given example of *E*_pass_ = 580 eV, the resulting bandpass width of 30–70 eV is sufficient to eliminate all unwanted energies from the spectrum. The final energy resolution is achieved in the ToF section. For this mode of operation, a diameter in the range of 0.5–1 mm for entrance and exit apertures is sufficient.

By reducing the pass energy and slit size, the dodecapole setup can also be used as a dispersive energy filter without subsequent ToF analysis. The present prototype with 4° deflection angle has been experimentally tested at pass energies and aperture sizes down to <20 eV and 100 µm, respectively. This combination results in a resolution well below 1 eV. It is crucial for this mode that a large fraction of the photoelectrons is focused into the small circular entrance aperture. This requires a high electron flux density emitted from the sample, *i.e.* a small photon footprint. On the other hand, the spherical aberration of lens group 1 must be minimized, in order to obtain a small image of the photon spot in the first Gaussian plane GP1. In the *k*-imaging mode, a maximum solid-angle interval is aimed at, which requires a large diameter of the *k* image in the first reciprocal image plane RP1 [see the left side of Fig. 3 ▸(*c*)].

The primary goal of this study was to develop an easy-to-use compact bandpass prefilter. We did not systematically vary the deflection angle α in the simulations. Nevertheless, the simulation revealed one basic property of this design: up to moderate angles, the dispersion increases linearly with α. Thus, the case shown in Fig. 3 ▸ but with α = 10° would already yield a resolution of 1% for a slit size of 200 µm. For *E*_pass_ < 50 eV, this would give a resolution of <500 meV, which fits well with the typical bandwidths of hard X-ray beamlines. The large *k* field of view is essentially retained, but the higher energy resolution at larger angles of α is paid for by a lower *k* resolution.

### 2.4. Implementation into a momentum microscope

**Figure 4:**
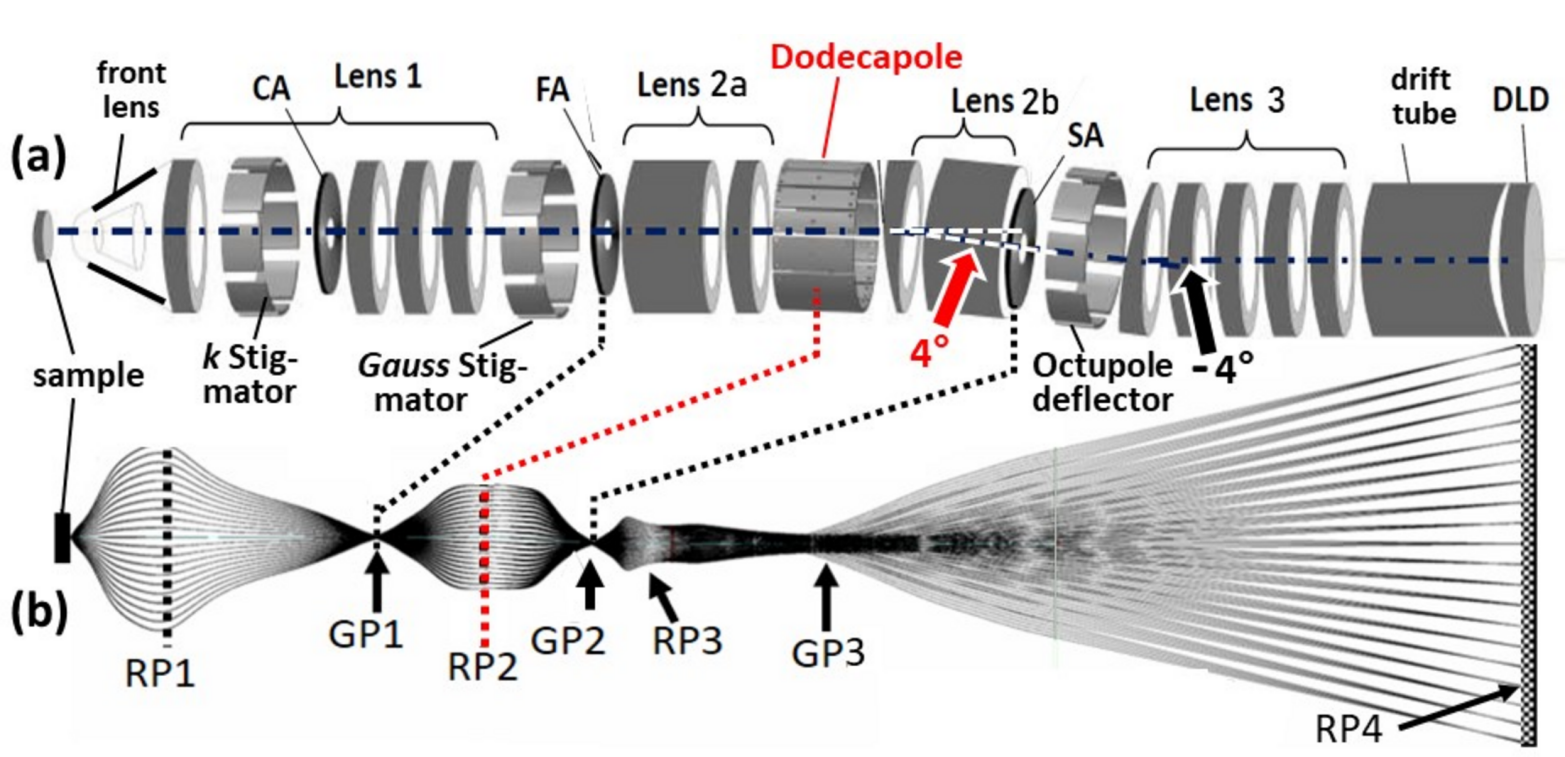
The ToF-MM with integrated dodecapole bandpass filter. (*a*) A schematic sketch with assignment of the elements. (*b*) Ray-tracing calculation. CA, FA and SA denote contrast aperture, field aperture and selector aperture, respectively; the latter two are piezomotor-driven arrays of 16 apertures of different sizes. GP and RP denote the Gaussian and reciprocal image planes. The optics also includes two octupole stigmators (close to RP1 and GP1) for beam shaping and an octupole deflector behind GP2 directing the beam parallel to the optical axis. DLD denotes the delay-line detector.

The dodecapole was implemented into a high-energy MM and installed at the hard X-ray beamline P22 of PETRA III (Kalha *et al.*, 2021 ▸). Fig. 4 ▸ shows a schematic view [Fig. 4 ▸(*a*)] and ray-tracing calculation [Fig. 4 ▸(*b*)] of the complete instrument. It comprises the front lens and four zoom-lens groups, as discussed in Figs. 1 ▸ and 3 ▸. Lens group 3 projects the *k* image (or alternatively a Gaussian image) onto the DLD via the ToF drift tube.

The optics comprises three octupoles serving as deflector/stigmator units, the first and second acting on the *k* and Gauss images and the third [behind the exit aperture (SA) of the dodecapole unit] for directing the beam parallel to the original optical axis. CA, FA and SA denote three sets of selectable and adjustable apertures: contrast aperture in the back-focal plane (reciprocal plane RP1), field aperture in the first intermediate image (in Gaussian plane GP1) and selector aperture (in plane GP2). The aperture arrays of the FA and the SA comprise auxiliary fine transmission electron microscopy grids for easy adjustment of the Gaussian planes. In the plane RP1, the *k* images have diameters of up to 20 mm, hence the CA grid is a hexagonal array of small holes.

In a modified setup we placed an array of CAs for PEEM mode in the conjugate plane RP2, where the size of the *k* image is smaller. The instrument can focus real-space or momentum images of the photoelectrons on the DLD, just by varying lens settings. For real-space imaging of a very large field of view up to >3 mm, the front lens is adjusted such that the first reciprocal image RP1 is shifted into the plane of the FA. Then, RP and GP are interchanged, and a real-space image is focused on the DLD. This is particularly important for ToF X-PEEM, *i.e.* spatial imaging on a core-level signal.

The front lens can be operated in different modes, as discussed by Tkach *et al.* (2024 ▸) and Tkach & Schönhense (2024 ▸). In the classical extractor mode, the first electrode is at a high positive voltage. In the zero-field mode, the extractor is ‘switched off’ by setting it equal to the sample potential. Finally, in the repeller mode, the first electrode is used as a retarding lens. This mode and, to some extent, the zero mode suppress the main part of the space-charge interaction with the slow electrons (Schönhense *et al.* 2021*b* ▸).

For a total deflection angle of α = 4° and a distance of 120 mm between the center of the dodecapole and the exit aperture [the scaled scheme in Fig. 3 ▸(*a*)], the beam displacement is just 8 mm. Such a small displacement is compatible with a linear vacuum vessel and a linear mumetal tube for magnetic shielding of the entire unit. Downstream of the exit aperture, a short octupole deflector/stigmator directs the beam by −4° parallel to the original optical axis. Here, the beam diameter is very small so that this second deflection is much less demanding than the deflection in the dodecapole, where a large *k* image is located.

## 3. Performance of the dodecapole bandpass filter in a time-of-flight momentum microscope using hard X-ray synchrotron radiation

### 3.1. Bandwidth as a function of pass energy and slit size

**Figure 5:**
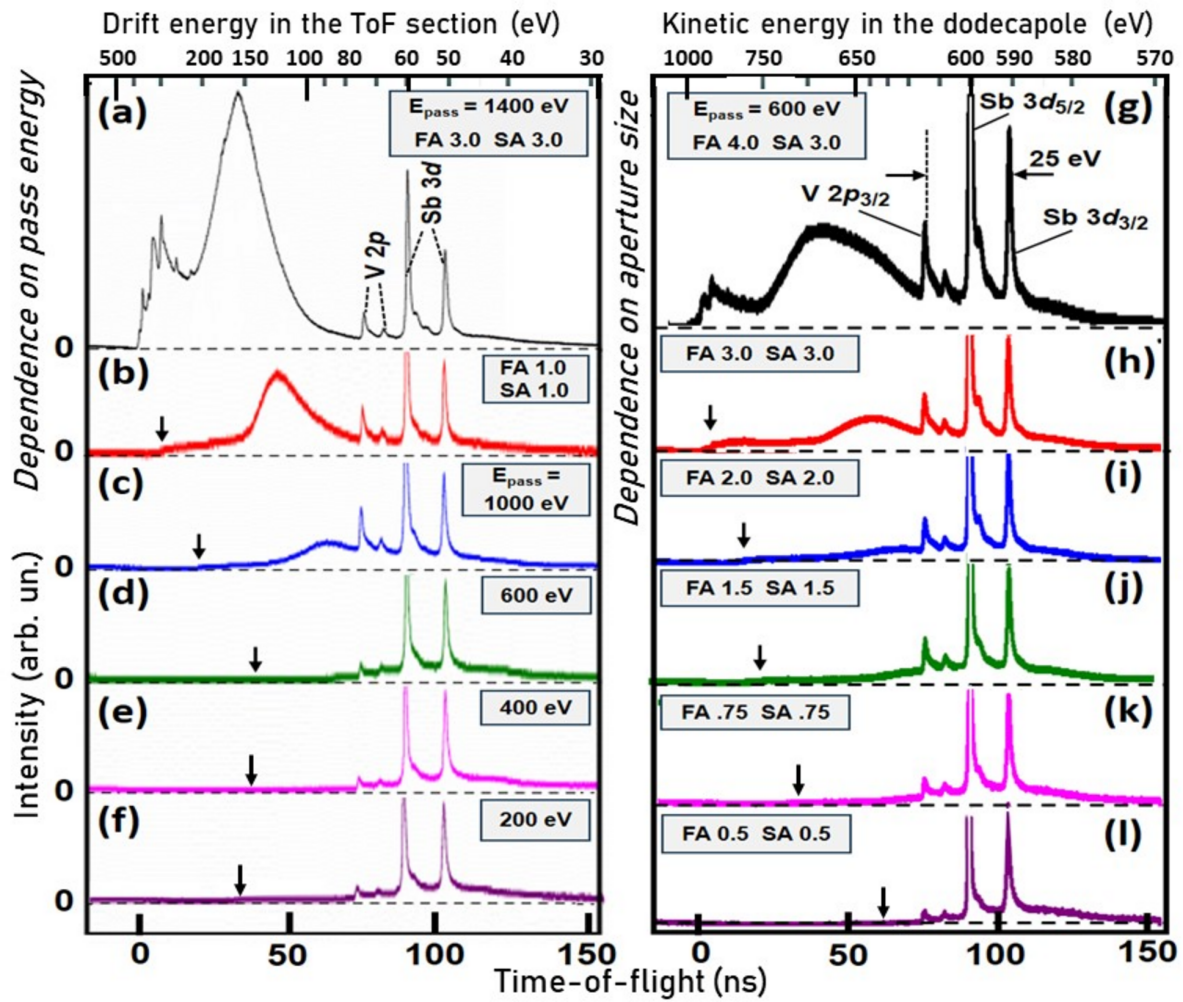
ToF spectra measured in the hard X-ray range (*h*ν = 5045 eV) for a series of pass energies of the dodecapole bandpass filter (left column), and for different entrance and exit apertures (right column). FA and SA denote field aperture (entrance ‘slit’) and selector aperture (exit ‘slit’), respectively; the numbers 4.0–0.5 give the diameters in millimetres. In order to emphasize the smaller signals, the maximum of the main peak Sb 3*d*_5/2_ is cut in panels (*b*)–(*l*). The non-linear scales at the top show the drift energy in the ToF section (left column) and the kinetic energy in the dodecapole (right column).

The performance of the setup shown in Fig. 4 ▸ was characterized at beamline P22 of PETRA III (DESY, Hamburg). The bandwidth selection was investigated at a photon energy of 5045 eV. Fig. 5 ▸ shows a series of ToF spectra for different pass energies and different slit sizes. A metallic film containing vanadium and antimony was used as a test sample. The photoelectrons from the Sb 3*d*_5/2_ core level were retarded to the pass energy *E*_pass_ of the dodecapole, as stated in the panels. Therefore, this core-level peak is at the center of the transmitted energy bands. The Sb 3*d*_5/2_ peak is framed on the low-energy side by its spin-orbit partner Sb 3*d*_3/2_ at a distance of 9 eV and on the high-energy side by the V 2*p* spin-orbit doublet, with a distance of 16 eV between the Sb 3*d*_5/2_ and V 2*p*_3/2_ peaks.

Figs. 5 ▸(*a*) and 5 ▸(*g*) show ToF spectra with peak assignments. The spectra are plotted as measured on a linear ToF scale. The corresponding kinetic energy scales are shown on top of the panels, reflecting the relationship *E*_kin_ ∝ τ^−2^ (τ = ToF). The entire group Sb 3*d* (*E*_B_ = 528/537 eV) plus V 2*p* (*E*_B_ = 512/520 eV) has a width of 25 eV, which is perfectly suited to study the confinement of the transmitted energy interval as a function of the pass energy and slit width. A number of further core levels of Sb and V at lower binding energies yield intense signals at higher kinetic energies, providing a rich scenario for observing the suppression of higher-energy electrons by the bandpass filter.

The left column of Fig. 5 ▸ shows a series of ToF spectra as a function of pass energy between *E*_pass_ = 1400 eV [Fig. 5 ▸(*a*)] and *E*_pass_ = 200 eV [Fig. 5 ▸(*f*)]. The non-linear scale at the top shows the kinetic energy in the drift tube of the ToF section. The spectrum transmitted by the dodecapole is recorded by the subsequent ToF analysis. Here, the Sb 3*d*_5/2_ peak is set to a drift energy of 60 eV. The first spectrum [Fig. 5 ▸(*a*)] was recorded with large slit sizes of 3 mm to visualize the high-energy part of the spectrum at flight times between 0 and 50 ns, corresponding to drift energies up to 500 eV. Indeed, the group of shallow core levels of the constituent atoms appears on the left side of the spectrum. At such high drift energies, the energy scale is strongly compressed; resulting in a total spectrum of 470 eV. By reducing the slit size to 1 mm (at *E*_pass_ = 1400 eV), the high-energy component is strongly suppressed, Fig. 5 ▸(*b*). Stepwise reduction of the pass energy removes all electrons faster than the desired energy interval [Figs. 5 ▸(*c*)–5 ▸(*f*)]. The high-energy onset of the spectra is indicated by arrows.

The right column of Fig. 5 ▸ shows the analogous series for varying the aperture size, recorded for *E*_pass_ = 600 eV. The non-linear scale at the top shows the kinetic energy in the dodecapole. For the large aperture diameters of 4 mm/3 mm, the spectrum extends into the region of the shallow core levels [Fig. 5 ▸(*g*)]. In the sequence of the slit sizes 3, 2, 1.5, 0.75 and 0.5 mm [Figs. 5 ▸(*h*)–5 ▸(*l*)], the spectrum is increasingly narrowed. The high-energy onset of the spectra is marked by arrows. In the 0.5 mm spectrum [Fig. 5 ▸(*l*)], the onset is at 630 eV and the intensity drops to zero at about 570 eV. Assuming a shape as shown in Fig. 3 ▸(*e*), we estimate an effective bandpass of the order of 20 eV (FWHM), in good agreement with the simulation. As expected, the leading edge is steeper at higher energies than the trailing edge at lower energies.

With transmitted bandwidths between 20 and several hundred electronvolts, the bandpass filter behaves as expected from the ray-tracing simulations in Fig. 3 ▸(*e*). In a first attempt at even lower pass energies with a laboratory source, we found bandpass widths of <1 eV for a polycrystalline sample (without *k* imaging).

### 3.2. Imaging properties of the dodecapole filter

It was expected that the spectral performance of the dodecapole filter would not deviate significantly from the theoretical expectation. The behavior of the electron beam in the largely homogeneous deflector field, Fig. 2 ▸(*b*), is reliably predicted by the simulation. Therefore, the measured values for the bandpass are in good agreement with expectations. However, it is not clear whether the imaging performance, in particular the fringing field effects at the entrance and exit, are correctly described in the 3D model in *SIMION* (Dahl *et al.*, 2007 ▸). As the beam passes through the small entrance and exit apertures, the *k* image is ‘encoded’ in the form of an angular pattern. Fringing fields can strongly affect this angular pattern. Extensive work has been carried out on the fringing fields of hemispherical analyzers. In a classic work, Jost (1979 ▸) introduced a special electrode geometry, and recently Tusche *et al.* (2019 ▸) discussed the fringing field effect of the ‘Jost electrodes’ for hemisphere-based MMs. Due to the small deflection angle α, our design of the dodecapole filter does not include electrodes for fringing field correction. However, until the first experiments, it remained unclear whether the dodecapole would lead to significant image aberrations.

**Figure 6:**
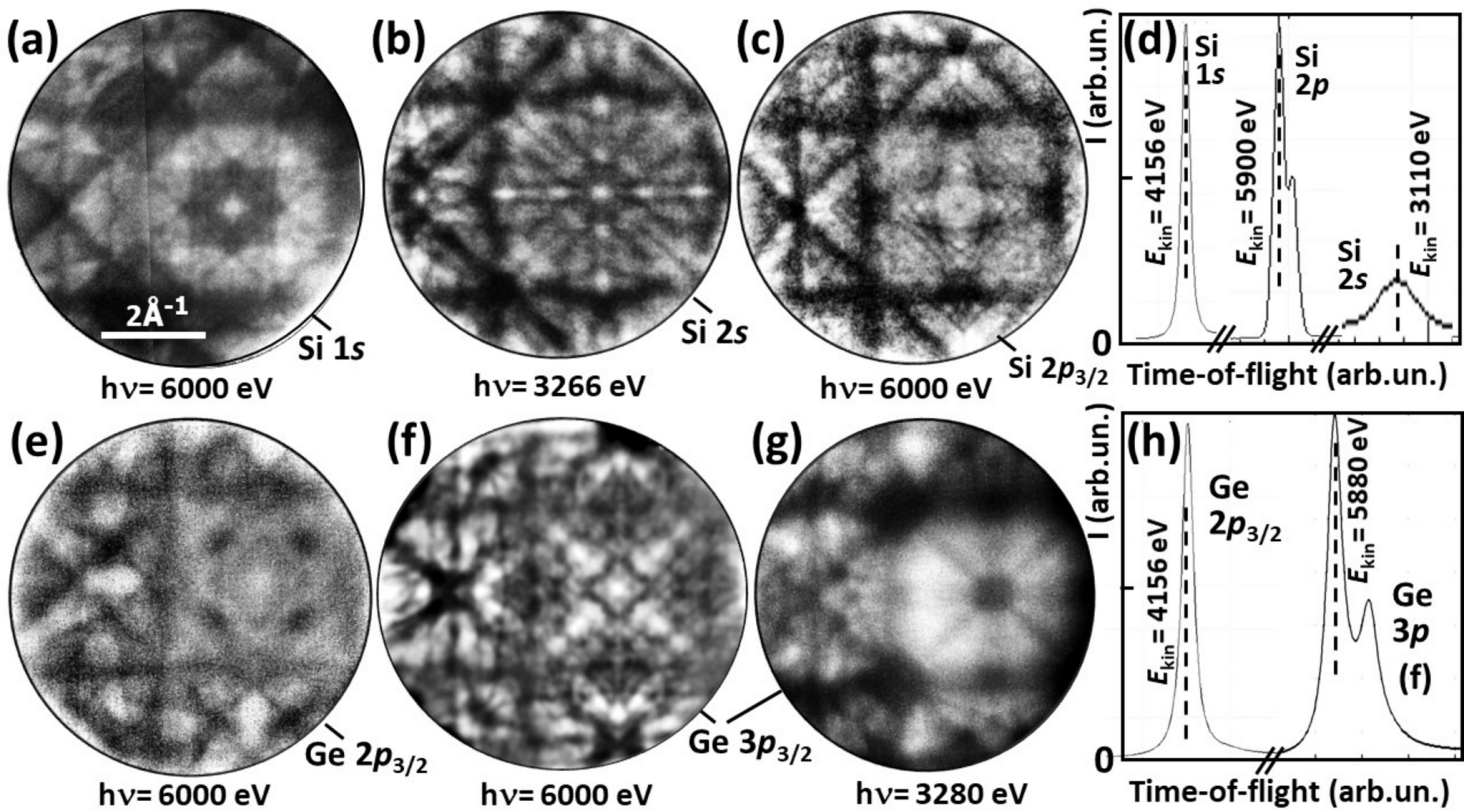
Hard X-ray core-level photoelectron diffraction patterns measured at beamline P22 of PETRA III. The top row shows silicon 1*s*, 2*s* and 2*p* patterns and the bottom row shows germanium 2*p* and 3*p* patterns, recorded at (*a*), (*c*), (*e*), (*f*) *h*ν = 6 keV, (*b*) 3.266 keV and (*g*) 3.28 keV. (*d*, *h*) Corresponding ToF spectra with corresponding energies *E*_kin_.

We demonstrate the imaging performance of the bandpass filter for two key applications of the high-energy MM at beamline P22: (i) full-field core-level photoelectron diffraction and (ii) bulk valence band (VB) mapping. Fig. 6 ▸ shows a selection of core-level diffractograms for Si 1*s*, 2*s* and 2*p* [Figs. 6 ▸(*a*), 6 ▸(*b*) and 6 ▸(*c*)], and Ge 2*p* and 3*p* [Figs. 6 ▸(*e*), 6 ▸(*f*) and 6 ▸(*g*)] with their corresponding ToF spectra [Figs. 6 ▸(*d*) and 6 ▸(*h*)]. The pass energy of the dodecapole was 400 eV. At these energies, the X-ray photoelectron diffraction patterns exhibit pronounced Kikuchi diagrams. These are rich in detail and show superior contrast and resolution compared with previous measurements on Si (Fedchenko *et al.*, 2020 ▸; Hoesch *et al.*, 2023 ▸), Ge (Tkach *et al.*, 2023 ▸) and GaAs (Medjanik *et al.*, 2021 ▸). We attribute this partly to the blocking of electron paths far from the optical axis combined with a 20–30% smaller *k* field of view and partly to the background reduction provided by the dodecapole prefilter. The latter effect is also visible in the spectra, which show virtually no background.

In these examples, the count rate in the DLD was >10^6^ counts per second. The photoelectron diffraction patterns could then be observed in real time at a frame rate of a few seconds. Without the bandpass filter, the background is much higher and the majority of electrons reaching the DLD do not come from the core level of interest. In turn, the saturation of the DLD limits the recording speed. One of the most important applications of full-field X-ray photoelectron diffraction using an MM is the structural and spectroscopic analysis of dopants in semiconductors – see examples of Mn in GaAs (Medjanik *et al.*, 2021 ▸) and Te in Si (Hoesch *et al.*, 2023 ▸). In these cases, the core-level signals of the dopants are quite weak, so the background suppression is even more important. However, the main advantage of the bandpass is the elimination of higher-order artefacts, such as those shown in Figs. 1 ▸(*e*) and 1 ▸(*f*). The final energy resolution is not affected by the dodecapole filter as it is defined by the time resolution and the drift energy in the ToF analyzer.

As an example of bulk VB mapping, we have chosen silicon. Despite its high Debye temperature, Θ_D_ ≃ 680 K, this light element (*Z* = 14) should not be suitable for hard X-ray ARPES (HARPES). The Debye–Waller factor is considered to determine the ratio of indirect to direct transitions. For Si, this ratio already reaches 0.5 at *h*ν ≃ 1 keV and 20 K, see Fig. 30 in the work of Fadley (2010 ▸). This regime is called the ‘XPS limit’ (Fadley, 2010 ▸) because phonon-scattering processes randomize the *k* information while almost preserving the energy, *i.e.* the XPS spectra.

**Figure 7:**
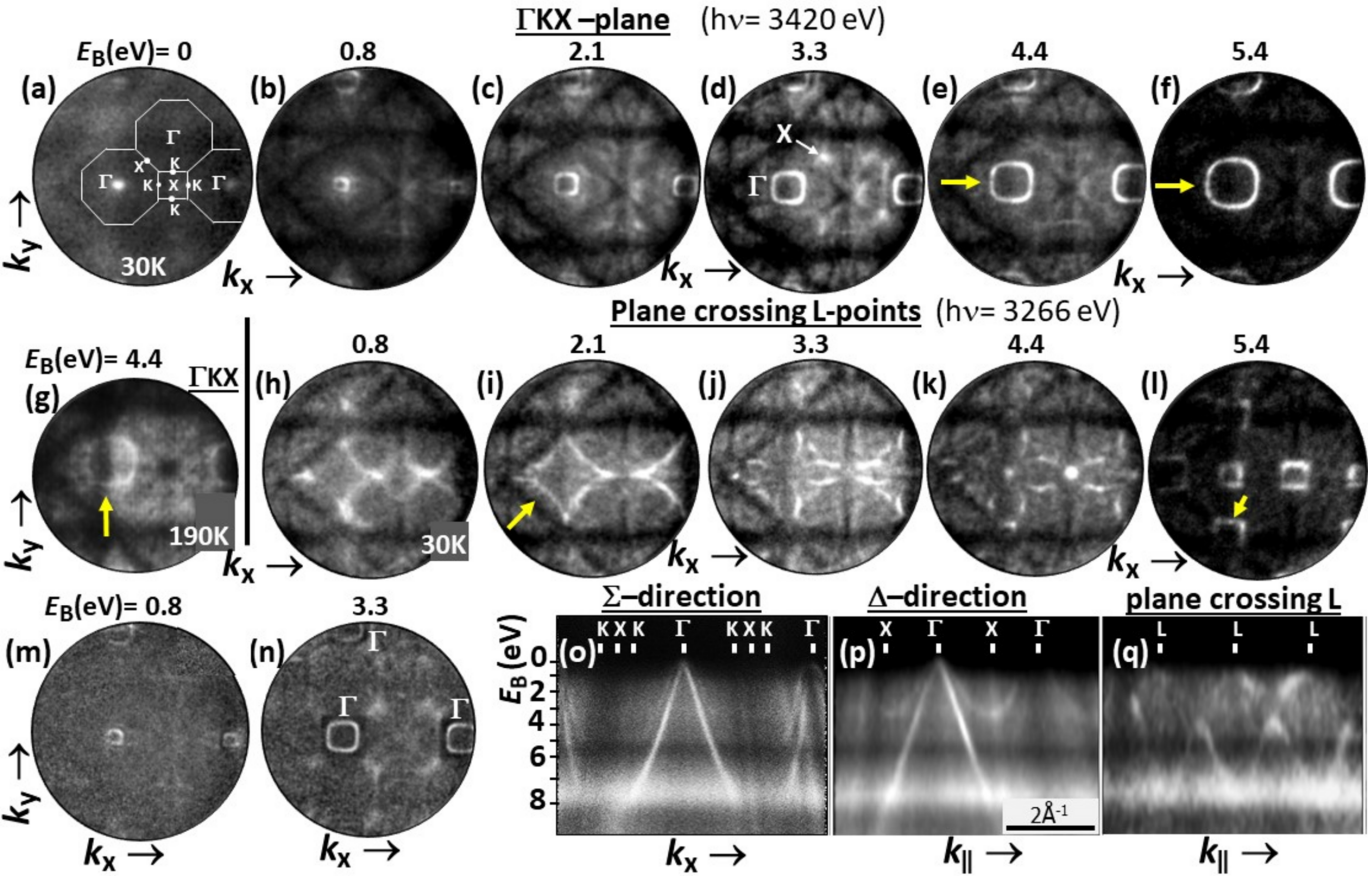
Hard X-ray VB patterns of Si(100), recorded at beamline P22 of PETRA III using a pass energy of the dodecapole of *E*_pass_ = 600 eV [sample temperature of 30 K, except for (*g*)]. (*a*)–(*f*) A series of *k*_*x*_–*k*_*y*_ patterns; *E*_B_ values are given on top of the panels. *E*_B_ is referenced to the VB maximum. At *h*ν = 3420 eV (top row), the direct transition leads to the ΓKX plane of the 13th repeated BZ along *k*_z_. (*g*) Same as (*e*) but for 190 K. (*h*)–(*l*) Same binding energies as (*b*)–(*f*) but at *h*ν = 3266 eV, leading to the plane intersecting the L points of the 3D BZ. (*m*), (*n*) Patterns (*b*) and (*d*) after eliminating the imprinted Kikuchi diffraction by multiplicative correction. (*o*)–(*q*) *E*_B_ vs *k*_∥_ cuts along the Σ and Δ directions, and in the plane through the L points. Apart from (*m*) and (*n*), all panels show raw data as measured.

Fig. 7 ▸ shows a sequence of *k*_*x*_–*k*_*y*_ sections for Si(001) recorded at *h*ν = 3420 eV [Figs. 7 ▸(*a*)–7 ▸(*g*)] and *h*ν = 3266 eV [Figs. 7 ▸(*h*)–7 ▸(*l*)]. These energies were chosen because in 3D *k* space the photoemission final-state sphere in the 13th repeated Brillouin zone (BZ) intersects the high-symmetry plane ΓKX (at 3420 eV) and the plane crossing the L points, halfway between Γ and the rim of the BZ (at 3266 eV). Here we only demonstrate the quality of the band structure features and discuss some peculiarities of hard X-ray photoemission of light elements; a more detailed analysis is in preparation (Schönhense *et al.*, 2024 ▸). The first row shows the VB patterns at binding energies (*E*_B_) between 0 and 5.4 eV (with respect to the VB maximum). The series shows the topmost band visible as a single dot in Fig. 7 ▸(*a*), opening to an outwardly dispersing band (fourfold symmetry) with increasing *E*_B_ in Figs. 7 ▸(*b*)–7 ▸(*f*). The dispersions along the Σ and Δ directions are shown in the *E*_B_ vs *k*_∥_ plots, Figs. 7 ▸(*o*) and 7 ▸(*p*). The uppermost band looks sharp up to its top, which defines the VB maximum. The *k*_*x*_–*k*_*y*_ patterns at the same *E*_B_ values in the second row [Figs. 7 ▸(*h*)–7 ▸(*l*)] look very different because the different photon energy leads to another plane in the 3D BZ that cuts through the L points. The corresponding *E*_B_ vs *k*_∥_ section in Fig. 7 ▸(*q*) shows that in this plane the bands do not reach the VB maximum. In Fig. 7 ▸(*p*) the split-off band with its minimum at the X point is clearly visible, all in agreement with calculations for Si (Chelikowsky & Cohen, 1974 ▸). In agreement with Strocov *et al.* (2023 ▸), we do not find any traces of multiband final states for Si.

All images show not only the bright dispersing band features but also a highly structured background consisting of a symmetrical pattern of distinct dark lines that show no dispersion, *cf*. Figs. 7 ▸(*a*)–7 ▸(*g*) and 7 ▸(*h*)–7 ▸(*l*). This background is caused by quasi-elastic phonon-scattering processes and has been observed and discussed in previous HARPES experiments. It has a characteristic spectral shape reflecting the matrix-element-weighted density of states (MEWDOS) (Fadley, 2010 ▸; Gray *et al.*, 2011 ▸, 2012 ▸; Nemšák *et al.*, 2016 ▸). The MEWDOS background is visible in the form of diffuse bright regions in the *E*_B_ vs *k*_∥_ plots – Figs. 7 ▸(*o*), 7 ▸(*p*) and 7 ▸(*q*). In electron microscopy, the origin of this background is called ‘thermal diffuse scattering’ and has been studied in detail (Wang, 2003 ▸, and references therein). The series in Figs. 7 ▸(*a*)–7 ▸(*l*) confirm our earlier observation for several elements and compounds (Babenkov *et al.*, 2019 ▸) that this background of indirect transitions is by no means ‘diffuse’ but shows a pronounced structure. A comparison of Figs. 7 ▸(*e*) and 7 ▸(*g*), both for *E*_B_ = 4.4 eV, but for sample temperatures 30 and 190 K, respectively, reveals that this background increases with increasing temperature, at the expense of the direct transitions. The electrons that ‘drop out’ of the coherent final-state wavefield due to a scattering process experience Kikuchi and Laue diffraction as they escape from the crystal; a detailed discussion is given by Schönhense *et al.* (2020*b* ▸).

Close inspection of the imprinted Kikuchi patterns reveals that the structured backgrounds in Figs. 7 ▸(*c*)–7 ▸(*e*) and 7 ▸(*h*)–7 ▸(*k*) are significantly different, reflecting the different wavelength of the electrons at the two energies. A qualitative comparison of Figs. 7 ▸ and 6 ▸ also shows that the richness in detail of the VB and core-level Kikuchi patterns is very similar. The VB patterns after multiplicative correction by the Kikuchi pattern are shown in Figs. 7 ▸(*m*) and 7 ▸(*n*); for details of this procedure, see Babenkov *et al.* (2019 ▸). Finally, the modulation by the Kikuchi pattern is even visible in the band features themselves, as marked by arrows in Figs. 7 ▸(*e*), 7 ▸(*f*), 7 ▸(*g*), 7 ▸(*i*) and 7 ▸(*l*). Fine dark Kikuchi lines modulate the VB ring in Figs. 7 ▸(*e*), 7 ▸(*f*), 7 ▸(*i*) and 7 ▸(*l*), and the light–dark transition at the edge of the primary rectangular Kikuchi band suppresses the intensity of the VB ring in Fig. 7 ▸(*g*). In agreement with DOS calculations (Askerov, 1994 ▸), the MEWDOS background shows a deep minimum between *E*_B_ = 5 and 6 eV, visible as a dark horizontal stripe in Figs. 7 ▸(*o*), 7 ▸(*p*) and 7 ▸(*q*). This has a very positive effect on the *k*_*x*_–*k*_*y*_ patterns at *E*_B_ = 5.4 eV, where the Kikuchi background is almost invisible. The band patterns show excellent contrast even without correction and their modulation by sharp dark Kikuchi lines is striking, see the arrows in Figs. 7 ▸(*f*) and 7 ▸(*l*).

## 4. Summary and conclusions

In ToF instruments, photoelectrons with higher energy than the electrons of interest can pass through the spectrometer and reach the detector, similar to a high-pass filter. If the ToF τ of the faster electrons differs by an integer multiple of the period *T* of the photon pulses, these electrons appear in the same time window as the true signal. The detector cannot distinguish electrons with τ differing by ±*nT* (*n* = a natural number). This phenomenon is commonly referred to as ‘temporal aliasing’. In momentum microscopy, artefact signals from this effect can be superimposed on the true signal (Medjanik *et al.*, 2019 ▸). Temporal aliasing is particularly severe in the X-ray region, where the energy spectrum is very broad and higher-order contributions from the monochromator and undulator release electrons with even higher energies. In addition to creating artefacts, such unwanted electrons can also lead to detector saturation.

Here, we described a compact bandpass prefilter that eliminates electrons with energies above or below the desired range before the beam enters the ToF analyzer. The heart of the device is an electrostatic dodecapole with an asymmetric electrode arrangement that allows energy-dispersive beam deflection and correction of image aberrations up to the third order. The electrode configuration is designed so that the energy-dispersive dipole field is homogeneous over a large part (∼80%) of the cross-sectional area. The filter is framed by transfer lenses in the input and output branches. Thanks to a beam deflection angle of only 4°, the entire instrument is very compact and housed in a straight mumetal vacuum tube. This type of background reduction also works with other types of analyzers (Schönhense & Schönhense, 2021 ▸). The filter can be integrated into MMs or PEEMs for imaging in real or reciprocal space.

We presented results for a dodecapole filter in a high-energy MM endstation at the hard X-ray beamline P22 at PETRA III. Systematic measurements for pass energies between 200 and 1400 eV and entrance and exit apertures between 0.5 and 4 mm (on two piezomotor-driven arrays with 16 apertures each) confirmed the expected spectral confinement, and recorded transmitted energy intervals between 20 eV and several hundred electronvolts. In this preselector mode, artefacts caused by higher-energy electrons, as well as slow secondary electrons, are effectively eliminated. In this beamline, the photon footprint is small, allowing a large fraction of the electrons within the desired energy band to pass through the entrance and exit apertures. These are the same criteria that have been discussed in the context of a hemispherical analyzer as a prefilter (Schönhense *et al.*, 2020*a* ▸, 2021*a* ▸).

In conclusion, an electrostatic dodecapole framed by two transfer lenses and arrays of entrance and exit apertures can serve as a simple but effective bandpass filter. For use as a prefilter in a ToF spectrometer, a deflection angle of 4° proved to be a good compromise, providing sufficient resolution, negligible aberrations and a small beam shift of a few millimetres (compatible with a straight vacuum housing). First tests with a low-energy laboratory source showed a bandpass of <1 eV, allowing spectroscopy without subsequent ToF analysis. Here, we have shown examples where the final energy resolution is defined by the ToF analysis. The electron-optical system shown in Fig. 4 ▸ enables momentum microscopy, full-field photoelectron diffraction and high-resolution X-PEEM [first results by Tkach *et al.* (2024 ▸)]. Sub-micrometre HARPES exploits the confinement of the probe spot by small FAs. The ultimate performance limit is posed by the space-charge interaction, which can be reduced by a novel front-lens architecture. The front lens of the instrument combines the conventional extractor mode (for maximum *k* field of view) with the zero-field mode (for 3D structured samples) and the repeller mode (for space-charge suppression) (Tkach *et al.*, 2024 ▸; Kalha *et al.*, 2021 ▸). The dodecapole improves the signal-to-background ratio and eliminates signals from higher-order admixtures in the photon beam. The instrument operates for kinetic energies up to *E*_kin_ > 8 keV.

## 5. Data availability

All data shown within this article are available on reasonable request.
